# Assessing Protein Immunogenicity with a Dendritic Cell Line-Derived Endolysosomal Degradome

**DOI:** 10.1371/journal.pone.0017278

**Published:** 2011-02-16

**Authors:** Matthias Egger, Alexander Jürets, Michael Wallner, Peter Briza, Silke Ruzek, Stefan Hainzl, Ulrike Pichler, Claudia Kitzmüller, Barbara Bohle, Christian G. Huber, Fátima Ferreira

**Affiliations:** 1 Christian Doppler Laboratory for Allergy Diagnosis and Therapy, Department of Molecular Biology, University of Salzburg, Salzburg, Austria; 2 Department of Molecular Biology, Division of Allergy and Immunology, University of Salzburg, Salzburg, Austria; 3 Department of Molecular Biology, Division of Chemistry and Bioanalytics, University of Salzburg, Salzburg, Austria; 4 Laboratory for Immunological and Molecular Cancer Research, Paracelsus Private Medical University of Salzburg, Salzburg, Austria; 5 Christian Doppler Laboratory for Immunomodulation, Department of Pathophysiology and Allergy Research, Medical University of Vienna, Vienna, Austria; Universidade de Sao Paulo, Brazil

## Abstract

**Background:**

The growing number of novel candidate molecules for the treatment of allergic diseases imposed a dramatic increase in the demand for animal experiments to select immunogenic vaccines, a pre-requisite for efficacy. Because no *in vitro* methods to predict the immunogenicity of a protein are currently available, we developed an *in vitro* assay that exploits the link between a protein's immunogenicity and its susceptibility to endolysosomal proteolysis.

**Methodology:**

We compared protein composition and proteolytic activity of endolysosomal fractions isolated from murine bone marrow- and human blood- derived dendritic cells, and from the dendritic cell line JAWS II. Three groups of structurally related antigen variants differing in their ability to elicit immune responses *in vivo* (Bet v 1.0101 and Bet v 1.0401, RNases A and S, holo- and apo-HRP) were subjected to *in vitro* simulated endolysosomal degradation. Kinetics and patterns of generated proteolytic peptides were evaluated by gel electrophoresis and mass spectrometry.

**Results:**

Antigens displaying weak capacity of T cell priming *in vivo* were highly susceptible to endolysosomal proteases *in vitro*. As proteolytic composition, activity, and specificity of endolysosomal fractions derived from human and murine dendritic cells were comparable, the JAWS II cell line could be used as a substitute for freshly isolated human or murine cells in *in vitro* degradation assays.

**Conclusions:**

Endolysosomal fractions prepared from the JAWS II cell line provide a reliable tool for *in vitro* estimation of protein immunogenicity. The rapid and simple assay described here is very useful to study the immunogenic properties of a protein, and can help to replace, reduce, and refine animal experiments in allergy research and vaccine development in general.

## Introduction

The development of a novel vaccine is a highly complex and demanding process that, from the initial concept to a licensed product, can take up to decades. Once a candidate has evolved in the laboratory, it undergoes vast series of pre-clinical *in vitro* and *in vivo* examination and optimization procedures. Evidently, only a minority of candidates passes all these obstacles, is permitted to clinical trials, accepted by regulatory agencies, and converted into a commercial product. The development of allergy vaccines faces additional problems, because unlike prophylactic vaccination, allergen-specific immunotherapy (SIT) attempts to counteract an already established pathological immune response [1]. Severe anaphylactic side effects can result from interactions between the administered vaccine and allergen-specific IgE antibodies of the atopic patient. Moreover, the current use of extracts of undefined contents can lead to sensitizations against new allergens during conventional immunotherapy [2]. Thus, allergy research today focuses on strategies to improve both, safety and clinical efficacy of SIT.

Others and we have proposed the substitution of allergen extracts in immunotherapy by molecule-based vaccines in order to implement safer and patient-tailored treatment [2], [3]. However, in contrast to infectious disease antigens, many allergens have been reported to be weak immunogens [4]–[7], a property hampering therapeutic success. Notably, it has been shown that allergen isoforms can differ by both immunogenicity (T cell reactivity) and allergenicity (IgE reactivity). For example, the birch pollen major allergen Bet v 1 isoform 0401 activates T cells much more efficiently than Bet v 1.0101 [8] but displays reduced IgE reactivity (hypoallergen) [4], [9], [10]. As molecules with such properties would bypass IgE mediated side effects during SIT, they are considered ideal allergy vaccines. Besides naturally occurring hypoallergens, modern DNA technology facilitates genetic engineering of recombinant hypoallergens [1]–[3], [11], [12]. However, the structural manipulation required for hypoallergen generation [11] and the production procedures [13] can severely affect the immunogenicity of a recombinant protein [14]–[17]. For example, although differing only by a single amino acid, one out of two *in silico* designed recombinant Bet v 1 mutants with reduced IgE reactivity lost its immunogenicity in mice [18]. Moreover, from 400 chimera generated by DNA shuffling of 14 major tree pollen allergens, only 2 fulfilled the requirements for efficient vaccine candidates [19]. The screening of such large candidate libraries requires high-throughput methods. Although IgE reactivity can be easily evaluated by antibody-based *in vitro* experiments in microtiter format, such tests are lacking for immunogenicity assessment. The prediction of T cell reactivity (immunogenicity) is costly, time-consuming, can only be performed for a limited number of molecules, and depends on *in vivo* or cell-based *ex vivo* systems. Nevertheless, ever since the first purified recombinant allergen was available in the early 1990s [20], a multitude of proteins has been produced as candidates for SIT [3], [11], [12]. Hence, the fast development of new molecule-based allergy vaccines dramatically increases the demand for animal sacrifice, conflicting the 3Rs declaration of the European Partnership for Alternative Approaches to Animal Testing (EPAA).

Within the present study we established a degradation assay based on earlier studies showing that susceptibility to endolysosomal proteolysis by antigen presenting cells (APC) serves as *in vitro* marker for protein immunogenicity [15], [16]. This assay enables the pre-selection of molecules with highest immunogenicity out of a big repertoire of related candidate proteins, hence aiming to replace, reduce, and refine animal experiments in allergy vaccine development. Whereas previous work solely focused on the kinetics of endolysosomal protein decomposition, we also evaluated the *in vitro* generated antigen-derived peptides and performed comparative fingerprinting of microsomal proteases. Moreover, we compared the degradative potential of different types of human and murine DC's microsomes. In addition to previously investigated antigens (*i.e.* ribonucleases A and S, as well as holo- and apo-horseradish peroxidase) [15], [16], we included two well-described allergens, *i.e.* the high and low allergenic isoforms of the birch pollen major allergen Bet v 1.0101 and Bet v 1.0401 [8], to evaluate the general applicability of the assay. As source for the endolysosomal hydrolases we employed a commercially available murine dendritic cell (DC) line [21]. This approach enables high-throughput screening for immunogenic candidates in vaccine development saving time, costs, human blood donors, and animal sacrifice, and might be interesting for any study on protein immunogenicity.

## Methods

### Subjects

All blood-donors gave written consent before enrollment in this study, which was approved by the local Medical Ethical Committee of Vienna.

### Mice

BALB/c mice were obtained from Charles River Laboratories (Wilmington, MA). All animal experiments were conducted according to National guidelines approved by the Austrian Ministry of Science (BMWF-66.012/0011-II/10b/2010).

### Antigens

Recombinant Bet v 1.0101 (SwissProt accession: P15497) and Bet v 1.0401 (SwissProt accession: P43177) were produced in *Escherichia coli* as recently published [8]. Ribonuclease (RNase A (SwissProt accession: P61823), RNase S, and holo-HRP (SwissProt accession: P00433) were purchased from Sigma-Aldrich, St. Louis, MO. Apo-HRP was prepared according to previously described protocols [15].

### Generation and culture of Dendritic Cells

Human mDCs (monocyte-derived DCs) obtained from heparinized blood samples and murine BMDCs (bone marrow-derived DCs) were generated and cultured as described elsewhere [8], [22]. The DC line JAWS II that has been established from bone marrow cells of a p53 knockout C57BL/6 mouse, was purchased from American Type Culture Collection (Manassas, VA) and cultured as previously described [21].

### Subcellular Fractionation and Fingerprinting of Microsomes

Endosomes and lysosomes were isolated from DCs by differential centrifugation [16]. Briefly, cells (10^7^ cells/protein) were homogenized in 10 mmol L^−1^ Tris/acetate pH 7 containing 250 mmol L^−1^ sucrose using a Dounce tissue grinder (Sigma-Aldrich, St. Louis, MO) and centrifuged for 10 minutes at 6,000× g. To obtain a total microsomal fraction, postnuclear supernatants were ultracentrifuged (60 minutes at 100,000× g). Microsomal content was released by 5 freeze-thaw cycles on liquid nitrogen and room temperature respectively, and stored at −20°C. For microsome fingerprinting 100 ng of microsomal proteins were reduced, alkylated, and trypsinized using the Calbiochem® ProteoExtract® All-in-One Trypsin digestion kit (EMD, Gibbstown, NJ) before injection into a one-dimensional capillary HPLC system (Model U3000, Dionex Benelux, Amsterdam, The Netherlands), equipped with a low-pressure gradient micro-pump, a micro-autoinjector and a capillary PS-DVB monolithic separation column (150×0.2 mm id). A 300 min gradient of 0–40% ACN in 0.05% aqueous TFA was applied using a flow rate of 1 µl min^−1^. The chromatographic setup was coupled to an ESI-LTQ Orbitrap mass analyzer (Thermo Fisher Scientific GmbH, Bremen, Germany). Each sample was analyzed in triplicate, while the peptides identified in one run were excluded from data dependent decisions in the following runs by the use of exclusion lists. Spectra were generated in positive mode in a mass range of m/z 500–2000. Fragmentation of a maximum of three precursors was realized in the ion trap using collision-induced dissociation. The Mascot search engine (Matrix Science, London, UK) and the software Proteome Discoverer (Thermo Fisher Scientific GmbH, Bremen, Germany) were used for peptide identification with the following parameters: taxonomy, all entries; Variable modification, methionine oxidation; fixed modification, carbamidomethyl (C), Enzyme, trypsin; peptide tolerance, ±10 ppm; MS/MS tolerance, ± 0.3 Da; maximum missed cleavages, 1; Human and murine samples were searched against species-specific SwissProt databases.

### Degradation Assays

Endolysosomal degradation assays were performed with 0.25 µg µl^−1^ of substrates (Bet v 1.0101, Bet v 1.0401, RNase A, RNase S, holo-HRP, apo-HRP) and 0.4 µg µl^−1^ of isolated microsomal proteins in a final volume of 20 µl containing 100 mmol L^−1^ citrate buffer pH 4.8 and 2 mmol L^−1^ dithiothreitol. Reactions were conducted for 0, 0.5, 1, 3, 5, 12, 24, 36, and 48 h at 37°C and stopped by boiling for 5 min at 95°C followed by freezing at −20°C. Alternatively, *in vitro* degradations were performed using 5×10^−4^ U µl^−1^ of purified human Cathepsin S purchased from Sigma-Aldrich, St. Louis, MO. Assays were quantitatively evaluated by flatbed scanner densitometry of GelCode® Blue Reagent (Thermo Scientific, Waltham, MA) stained sodium dodecyl sulfate polyacrylamide gels [23]. Qualitative analysis was performed by mass spectrometry using an ESI-QTOF mass spectrometer fitted with a capillary reversed phase HPLC (Waters, Milford, MA) as described elsewhere [22].

## Results

### Human and Murine DC-Derived Microsomes Display Comparable Endolysosomal Degradomes

To conduct endolysosomal *in vitro* degradation assays we isolated total microsomal (endolysosomal) fractions from human mDCs, murine BMDCs, and the murine JAWS II DC line employing a differential centrifugation protocol. Endolysosomal total proteomes were analyzed by mass spectrometry-based fingerprinting. 600 different proteins were detected in the microsomal fractions of all three different DC samples. Proteins belonging to the endolysosomal degradome or that have been shown to be involved in antigen processing are listed in Table 1. Cathepsins A, B, C, D, L, S, and Z as well as lysosomal prolylcarboxypeptidase and tripeptidyl peptidase 1 could be identified in all microsomal fractions. By contrast, cathepsin K, lysosomal dipeptidyl peptidase 2, and asparagine endopeptidase (AEP) were not detectable in BMDCs, and cathepsin H was not measured in the DC line. In summary, JAWS II DCs contain all important lysosomal endo- and exopeptidases that have been shown to be involved in antigen processing [24], [25]. Besides proteases, endolysosomal fractions also contained a multitude of other molecules (non-proteolytic acidic hydrolases, membrane-associated proteins, and GTPases involved in vesicular trafficking) that are associated with endosomal and lysosomal compartments of APCs (Table 1).

**Table 1:** Mass spectrometry based fingerprinting of DC-derived endolysosomal fractions.

Protein	mDC		BMDC		JAWS II		SwissProt accession	
	pep.	%	pep	%	pep	%	human	murine
**Endoproteases**								
Cathepsin D	20	60.44	16	59.76	20	63.17	P07339	P18242
Cathepsin K	-	-	-	-	6	41.03	-	P55097
Cathepsin L	4	29.73	7	42.81	2	15.87	P07711	P06797
Cathepsin S	13	48.94	9	54.12	10	47.94	P25774	O70370
AEP (legumain)	-	-	4	23.45	2	10.11	-	O89017
**Endo- and Exoproteases**								
Cathepsin B	21	54.57	17	55.16	12	47.79	P07858	P10605
Cathepsin H	7	35.22	6	27.93	-	-	P09668	P49935
**Exoproteases**								
Cathepsin A	7	24.38	16	36.50	15	41.98	P10619	P16675
Cathepsin C	13	41.25	10	29.22	11	32.47	P53634	P97821
Cathepsin Z	9	41.25	12	50.33	13	49.67	Q9UBR2	Q9WUU7
Dipeptidyl peptidase 2	-	-	2	6.32	3	9.88	-	Q9ET22
Prolylcarboxypeptidase	6	16.73	4	21.59	3	10.59	P42785	Q7TMR0
Tripeptidyl peptidase 1	7	25.93	6	23.31	5	23.21	O14773	O89023
**Non-proteolytic acidic hydrolases**								
Acid lipase	11	37.84	6	21.66	7	29.22	P38571	Q9Z0M5
Acid phosphatase	3	7.8	4	12.77	4	13.71	P11117	P24638
Alpha glucosidase	13	25.32	6	11.54	7	13.85	P10253	P70699
Alpha mannosidase	20	27.1	24	34.85	2	3.16	O00754	O09159
**Endolysosomal membrane-associated proteins**								
Clathrin	54	49.91	59	55.34	46	46.93	Q00610	Q68FD5
LAMP 1	4	9.59	7	17.98	5	14.53	P11279	P11438
LAMP 2	5	11.22	4	9.4	3	7.95	P13473	P17047
LMP 2	3	10.46	3	12.97	4	16.74	Q14108	O35114
VAMP 7	3	26.82	2	18.64	-	-	P51809	P70280
**GTPases involved in vesicular trafficking**								
ARF 6	6	49.14	5	44.57	-	-	P62330	P62331
Rab 5a	3	20.93	3	16.28	4	27.44	P20339	Q9CQD1
Rab 5c	9	66.20	9	67.13	7	61.11	P51148	P35278
Rab 7a	14	57.97	13	72.46	11	64.73	P51149	P51150

%, percentage to which identified peptides cover the full length protein sequence; AEP, Asparagine endopeptidase; ARF, ADP-ribosylation factor; LAMP, Lysosome-associated membrane glycoprotein; LMP, lysosome membrane protein; pep., identified peptides; Rab, Ras-like protein; VAMP, Vesicle-associated membrane protein.

### Kinetics of Endolysosomal Degradation Differ Between Structural Variants of the Same Antigen

**Figure 1:**
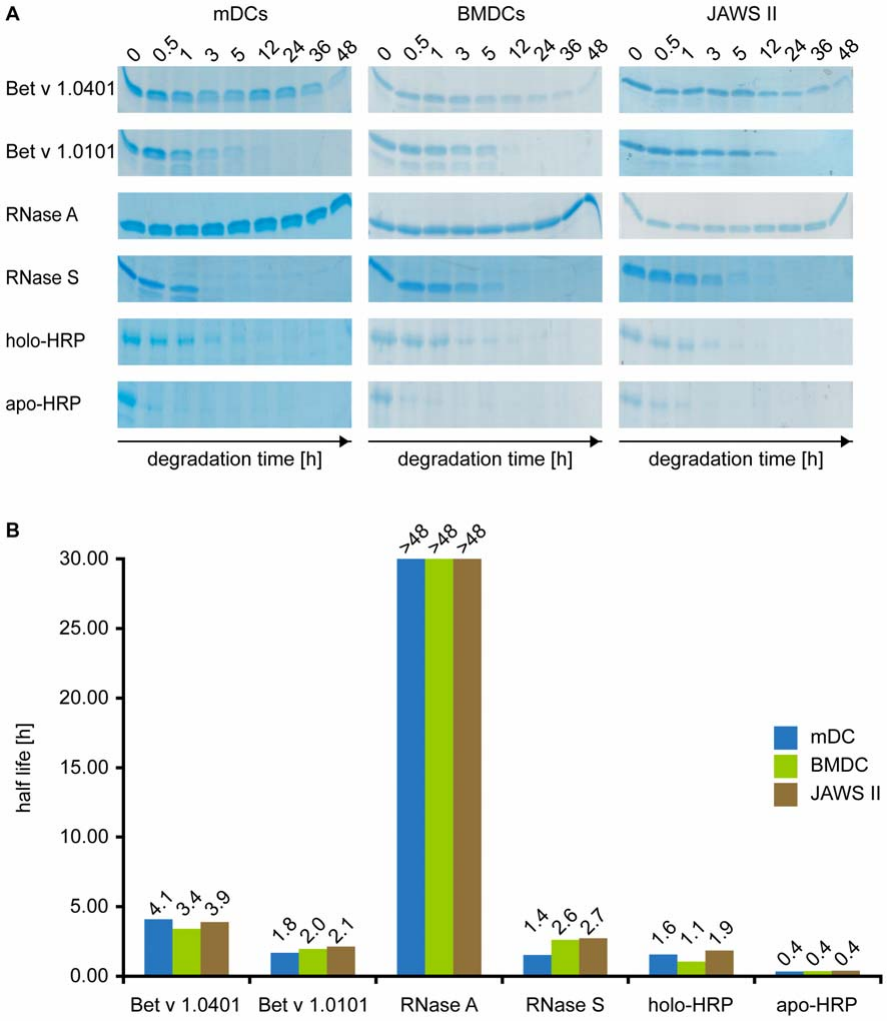
Kinetics of endolysosomal proteolysis. 2.5 µg of protein samples were analyzed by SDS-PAGE and GelCode® Blue staining after 0, 0.5, 1, 3, 5, 12, 24, 36 and 48 h of *in vitro* degradation using endolysosomal fractions isolated from human mDCs, murine BMDCs, and the DC line JAWS II (A). For each protein, the half-life during endolysosomal proteolysis was calculated from scanned and densitometrically quantified protein bands (B).

We compared the kinetics of endolysosomal decomposition for three pairs of antigens, *i.e.* structural variants of horseradish peroxidase (HRP), ribonuclease (RNase), and the major birch pollen allergen Bet v 1. The comparison of the average half lives (given in parenthesis) during endolysosomal *in vitro* degradation revealed that Bet v 1.0401 (3.8 h), RNase A (>48 h), and holo-HRP (1.5 h) were more resistant to proteolysis than Bet v 1.0101 (2 h), RNase S (2.2 h), and apo-HRP (0.4 h), respectively (Figures 1A and 1B). Thus, the decomposition of Bet v 1.0401 was around two-fold slower than observed for Bet v 1.0101. For the holo/apo-HRP and RNase A/S pairs endolysosomal half-life ratios were 3.8 and >20, respectively.

### Structural Variants of the Same Antigen Show Similar Patterns of Proteolytic Fragments

**Figure 2:**
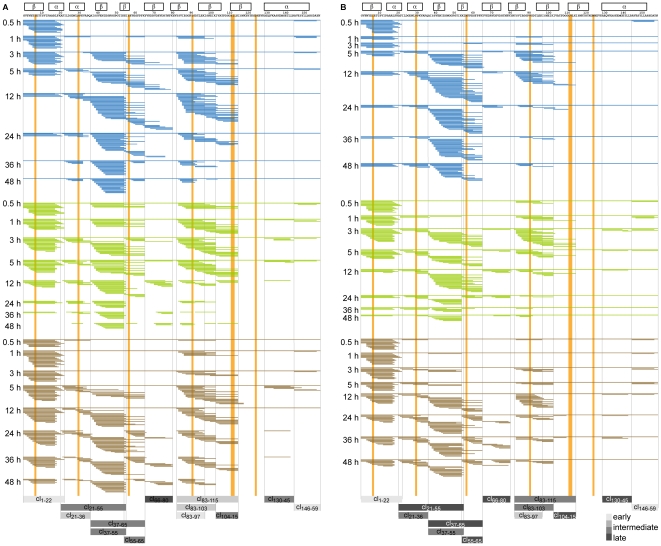
Chronology of Bet v 1 peptide cluster formation during endolysosomal proteolysis. Peptides sequenced by mass spectrometry after 0.5, 1, 3, 5, 12, 24, 36, and 48 h of *in vitro* digestion with microsomal fractions from mDCs (blue), BMDCs (green) and JAWS II DCs (brown) are shown for Bet v 1.0101 (A) and Bet v 1.0401 (B), respectively. Regions of predominant peptide clusters (cl_x-x_) are highlighted as bars colored in different shades of grey depending on their temporal occurrence (average appearance of early clusters: ≤1 h, intermediate clusters: >1 h and <5 h, and late clusters: ≥5 h. Bet v 1 secondary structures (α-helices and β-sheets) are indicated as framed boxes. Amino acids (n = 7) that differ between the 2 Bet v 1 isoforms are highlighted in orange.

**Figure 3:**
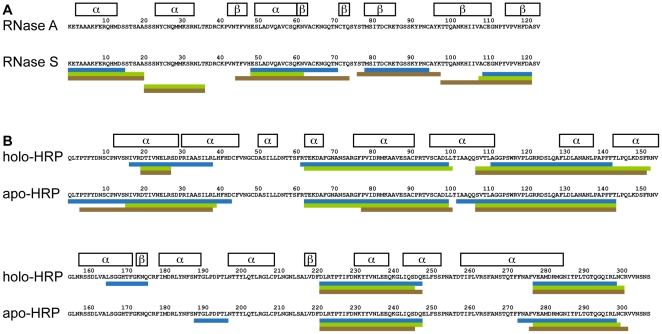
Regions of peptide clusters generated by endolysosomal proteolysis. Peptides sequenced by mass spectrometry after 0.5, 1, 3, 5, 12, 24, 36, and 48 h of *in vitro* digestion with microsomal fractions from mDCs (blue), BMDCs (green) and JAWS II DCs (brown) clustered in distinct regions along the protein sequence of RNase (A) and peroxidase (B) model antigens. Secondary structures (α-helices and β-sheets) are indicated as framed boxes.

Mass spectrometry-based analysis showed that peptides generated by endolysosomal *in vitro* proteolysis formed nested clusters sharing a common central core but displaying variable flanking regions. These features are characteristic for MHC (major histocompatibility complex) class II-bound peptides [22], [26]. Notably, peptides derived from both Bet v 1.0101 and Bet v 1.0401 were of 5 to 30 amino acids in length and clustered into 13 different regions along the Bet v 1 sequence. However, central (region Bet v 1_21–65_ and Bet v 1_83–115_) and C-terminal (region Bet v 1_130–159_) peptide clusters of the more stable isoallergen Bet v 1.0401 appeared temporally delayed. Interestingly, amino acids differing between the two Bet v 1 isoforms were rather located apart from proteolytic cutting sites in central regions of the peptide clusters (Figures 2A and 2B). Proteolytic fragments from both holo- and apo-HRP clustered in 5 analogous regions of the full-length molecules. Due to its high resistance to endolysosomal hydrolases, we could not detect any peptides from RNase A, whereas proteolytic fragments from RNase S were found in 5 different regions (Figure 3). According to our observations, differences in proteolytic resistance between pairs of antigens with similar sequence and conserved fold do not translate into altered patterns of proteolytic fragments.

### Endolysosomal Degradation of Bet v 1 is Mediated by Cathepsin S

**Figure 4:**
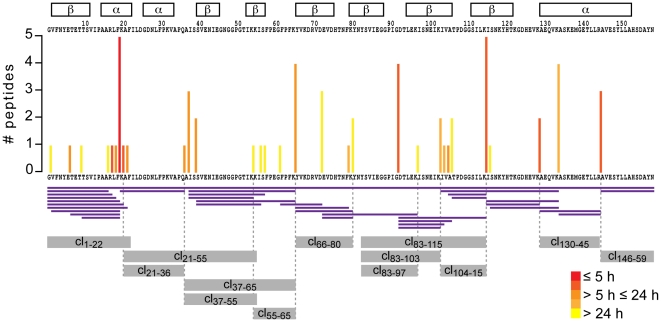
Cathepsin S digest of Bet v 1. Cathepsin S cleavage sites are depicted in the bar diagram showing their location in the Bet v 1.0101 sequence (*x-axis*) and the number of different peptides generated per cleavage site (*y-axis*). According to their temporal accessibility, cathepsin S sites are highlighted in red (early), orange (intermediate), and yellow (late) in the bar diagram. Generated peptides are shown as purple lines. Bet v 1.0101 secondary structures (α-helices and β-sheets) are indicated as framed boxes. Regions of Bet v 1.0101 peptide clusters (cl_x-x_) generated by endolysosomal proteases isolated from DCs are depicted as grey boxes.

Strikingly, the majority of *in vitro* generated Bet v 1-derived peptide clusters (Figure 2) was flanked by lysine residues (K20, 54, 55, 65, 80, 97, 103, 115, and 129). This observation suggests that cathepsin S, a lysosomal cysteine endoprotease favoring substrates with glutamines or lysines at the P1 position [27], could be involved in Bet v 1 decomposition. To pursue this idea, we performed *in vitro* degradation assays of Bet v 1.0101 with purified cathepsin S, and identified 31 cathepsin S sites. As shown in Figure 4, cathepsin S sites seem to be progressively accessible by the protease in a time-dependent manner. Notably, 8 out of the 13 Bet v 1.0101 peptide clusters that appeared during endolysosomal proteolysis applying microsomal fractions could also be generated by cathepsin S alone (Figure 4). Initially occurring Bet v 1.0101 peptide clusters contained (cl_1–22_) or were flanked (cl_146–59_) by early-cleaved cathepsin S sites (Figure 4). Short-time degradation assays with human microsomes (data not shown) revealed that the N-terminal cluster cl_1–22_ appeared already after a few minutes of reaction. Thus, cathepsin S might be engaged in the initial steps of Bet v 1.0101 processing. Late-appearing peptide clusters, which corresponded to the middle region of the Bet v 1 sequence (cl_83–115_, cl_83–103_, and cl_83–97_), might be produced by cathepsin S and AEP, a cysteine endopeptidase that strictly cleaves on the C-terminal site of asparagine residues (e.g. Bet v 1.0101 N82) [24], [25].

## Discussion

The ability to efficiently induce an immune response is a most important quality of any protein vaccine. As no *in vitro* tools have been available so far, immunogenicity is usually assessed *in vivo* by animal experiments or by *ex vivo* cell-based systems. The immunogenicity of an antigen strongly depends on the character of its interaction with DCs. These specialized APCs constitute the interface between antigens and adaptive immunity. After internalization, DCs convert antigen into peptides that are bound to MHC class II molecules, transported to the cell surface, and presented to CD4^+^ T helper cells. Of note, there is evidence that various subsets of DCs differ by their capacities of antigen processing *in vivo* [28]. For instance, the CD8^+^ DEC205^+^ subset of murine splenic DC is more biased for cross-presentation on MHC class I molecules, whereas the CD8^−^ DCIR2^+^ DC subset is specialized for MHC class II antigen presentation and displays increased expression of proteins involved in the exogenous antigen processing pathway, including cathepsins C, H, Z, and AEP. Further, it has been shown that compared to monocyte-derived DCs, conventional DCs are much more efficient in antigen processing and presentation independent from the route of antigen uptake [29].

Within the course of antigen processing, several steps (antigen uptake, DC activation, peptide generation, MHC-peptide complex stability and density) can be decisive regarding the immunogenicity of a given antigen. For instance, the internalization of ovalbumin conjugated to trimethyl chitosan by DCs was 5-fold increased leading to a 1,000-fold higher ovalbumin-specific IgG titers in immunized mice [30]. Apart from such adjuvant-dependent aspects, also properties intrinsic to the antigen (posttranslational modifications, structural features, and stability) can influence immunogenicity. For example, targeting receptor-mediated endocytosis, mannosylated ovalbumin induced a much stronger proliferation of ovalbumin-specific T cells than its unglycosylated counterpart [31]. Moreover, although structural discrepancies between isoforms of the birch pollen major allergen are minor (PDB accession numbers are given in parenthesis), uptake and endocytosis of Bet v 1.0101 (PDB: 1BV1) by murine BMDCs were significantly slower and less efficient than observed for Bet v 1.0401 (PDB: 3K78). The two isoforms also induced divergent patterns of DC activation and humoral responses, which have been attributed to cysteine-mediated dimerization due to a serine to cysteine exchange in Bet v 1.0401 [8]. In more detail, mice immunized with Bet v 1.0401 displayed a comparable IgE but a strongly elevated IgG and IgA response. On DCs Bet v 1.0401 elicited a significantly increased expression of CD80 and CD86 activation markers, whereas the secretion of cytokines antagonizing DC maturation and activation (IL-6) was enhanced in BMDCs stimulated with Bet v 1.0101. Notably, insufficient DC activation might be generally responsible for a shift towards the allergic immune response [32] and could in part explain hypoallergenicity of Bet v 1.0401.

Antigen uptake and DC activation might also affect the generation of T cell stimulatory peptides, a complex process that involves the sequential action of acidic hydrolases in endosomal and lysosomal compartments of the DC [24], [33]. Antigens that are rather stable to endolysosomal proteolysis can persistently provide peptides for binding to MHC molecules, thus ensuring efficient presentation to T cells and thus, high immunogenicity. By contrast, instable molecules fail to elicit an immune response due to rapid T cell epitope destruction [15], [16]. Hence, efficient antigen uptake of rather stable antigens might facilitate continuous delivery of peptides that can properly bind the MHC class II grove, thereby favoring a high density of MHC-peptide complexes on the surface of the DC for T cell presentation. Notably, the density of T cell epitopes is crucial for T cell activation [34] and could even be decisive in the development of a Th1 or allergic Th2 biased response [35]. Apart from such quantitative aspects, also the quality of peptides generated by endolysosomal proteases has a strong impact on the immunogenicity of proteins. For example, stabilization of MHC-peptide complexes by substituting single amino acids in a tumor antigen-derived T cell epitope increased specific T cell responses up to 50-fold [36]. Hence, the 7 amino acid exchanges in the Bet v 1.0401 isoform might also contribute to its stronger immunogenicity.

In the present study, we exploited the link between resistance to endolysosomal proteolysis and immunogenicity to establish a high-throughput screening procedure for rational protein vaccine development. Although the capacity of an antigen to induce an immune response depends on many parameters, according to our data susceptibility to endolysosomal proteases seems to be a key factor determining immunogenicity. Nevertheless, as cell-free endolysosomal *in vitro* degradations do not encompass important parameters of immunogenicity like antigen uptake, DC activation, and stability of the MHC-peptide complex, it cannot reflect the complex situation of antigen processing *in vivo*. Therefore, this method cannot be used for T cell epitope determination, and assessments on protein immunogenicity might deviate in some cases from *in vivo* obtained data. Still, the assay described here represents an excellent tool requiring only standard laboratory equipment, a tissue homogenizer, an ultracentrifuge, and a cell culture facility for rapid and simple assessment of protein immunogenicity. Because costs, time, up-scalability, and standardization problems limit the use of human as well as animal-derived DCs, we introduced the murine commercially available DC cell line JAWS II as source for endolysosomal proteases. Comparative mass spectrometry-based analysis of the protein composition provided strong evidence of the similarity between the different degradomes. To our knowledge, this represents the first study on the endolysosomal proteome from mice and men. We found that the proteome data nicely correlated with degradative activity and proteolytic specificity of the degradome using RNase A, HRP, and Bet v 1.0101 model antigens as substrates. Because structural features of proteins seem to be intimately connected to their immunogenic activity, as demonstrated earlier for structural variants of RNase A, HRP [15], [16], and Bet v 1 [8], we analyzed the kinetics and degradation patterns of RNase S, apo-HRP and Bet v 1.0401. Indeed, we observed remarkable differences in the proteolytic resistance and kinetics of degradation among the investigated antigens. It has been demonstrated that local structural flexibility is a general requirement for proteolytic sensitivity and that deletion of such elements enhances protein stability [37]. However, we did not observe a strict correlation between proteolytic cutting sites and loop structure elements in our model antigens and variants thereof. Of note, the flexible C-terminal loop of Bet v 1 (Bet v 1_123–129_) seems to be heavily degraded by lysosomal proteases, as we could not detect any *in vitro* generated peptides corresponding to this region. Thus, deletion of Bet v 1_123–129_ might increase Bet v 1.0101 stability and immunogenicity. Another possibility would be the targeting of frequently used and surface-exposed cathepsin S cleavage sites of Bet v 1.0101 (F19-K20, K65-Y66, G92-D93, K115-I116, and K134-A135) identified here.

Other factors influencing proteolytic sensitivity and immunogenicity are aggregation [8], integrity of the polypeptide backbone, and the presence of ligands [15], [16]. As described above, due to a serine to cysteine exchange at position 112, Bet v 1.0401 shows a high tendency to form disulfide-linked aggregates [8]. Presumably due to steric hindrance of proteolytic cutting sites, this isoform appears twice as stable in endolysosomal *in vitro* degradation assays than Bet v 1.0101. Accordingly, antibody and T cell responses are elevated in mice immunized with Bet v 1.0401. Similarly, RNase A induces more than 10,000-fold stronger T cell and IgG responses than RNase S, which is destabilized by a single subtilisin-mediated peptide bond cleavage between A20 and S21. During endolysosomal *in vitro* proteolysis RNase S was degraded more than 20-fold faster than RNase A. Moreover, apo-HRP lacking Ca^2+^ ions and the prosthetic heme group is a weak immunogen and displayed about 4-fold stronger sensitivity to endolysosomal proteases during *in vitro* degradation than fully equipped holo-HRP [15], [16].

In summary, our data convincingly showed that not only the proteome fingerprint but also the proteolytic activity in terms of specificity and kinetics of the analyzed degradomes is equivalent. Our findings are further supported by degradation experiments of Bet v 1.0101 using purified cathepsin S, an important endolysosomal protease. Remarkably, the patterns obtained by degradation with cathepsin S alone resembled those observed in reactions applying endolysosomal fractions. Despite the advantage of straightforward determination of proteolytic epitopes, the single protease approach cannot reliably mimic the complexity of a whole degradome since concerted action of proteases might be crucial in the kinetics of antigen degradation. As kinetics is the key indicator for assaying immunogenicity, this strategy would lack general applicability.

To conclude, the adoption of the JAWS II DC line for endolysosomal *in vitro* degradation facilitated the development of a simple, rapid, reliable, and easily upscalable comparative assay to select highly immunogenic allergy vaccine candidates from a pool of related molecules for pre-clinical evaluation. Data can be obtained within one week, and RNases A and S might serve as internal references. The method described here is very useful to study the immunogenic properties of a protein, and can help to replace, reduce, and refine animal experiments in allergy research and vaccine development in general.
